# Comparative Study of Ablation Zone of EMPRINT HP Microwave Device with Contemporary 2.4 GHz Microwave Devices in an Ex Vivo Porcine Liver Model

**DOI:** 10.3390/diagnostics13162702

**Published:** 2023-08-18

**Authors:** Terrence C. H. Hui, Guo Yuan How, Michelle S. M. Chim, Uei Pua

**Affiliations:** 1 Department of Diagnostic Radiology, Tan Tock Seng Hospital, Singapore 308433, Singapore; 2 Yong Loo Lin School of Medicine, National University of Singapore, Singapore 117597, Singapore

**Keywords:** percutaneous thermal ablation, microwave ablation, hepatocellular carcinoma, liver tumours, ex vivo bovine liver

## Abstract

(1) Background: Percutaneous microwave ablation (MWA) is an accepted treatment of non-operative liver cancer. This study compares the ablation zones of four commercially available 2.45 GHz MWA systems (Emprint, Eco, Neuwave, and Solero) in an ex vivo porcine liver model. (2) Methods: Ex vivo porcine livers (*n* = 85) were obtained. Two ablation time setting protocols were evaluated, the manufacturer’s recommended maximum time and a 3 min time, performed at the manufacturer-recommended maximum power setting. A total of 236 ablation samples were created with 32 (13.6%) samples rejected. A total of 204 samples were included in the statistical analysis. (3) Results: For single-probe protocols, Emprint achieved ablation zones with the largest SAD. Significant differences were found in all comparisons for the 3 min time setting and for all comparisons at the 10 min time setting except versus Neuwave LK15 and Eco. Emprint produced ablation zones that were also significantly more spherical (highest SI) than the single-probe ablations from all other manufacturers. No statistical differences were found for ablation shape or SAD between the single-probe protocols for Emprint and the three-probe protocols for Neuwave. (4) Conclusions: The new Emprint HP system achieved large and spherical ablation zones relative to other 2.45 GHz MWA systems.

## 1. Introduction

In the liver, percutaneous tumour ablation plays an important role in the management of very-early-stage and early-stage hepatocellular carcinomas (HCC) and hepatic metastases, offering less pain, shorter hospital stay, faster recovery and fewer complications compared to surgery [1,2,3].

Akin to surgery, the goal of percutaneous ablation is to cause necrosis of all tumour cells with margin while minimising damage to non-target tissue. Assuming most tumours are round, the ideal ablation zone is one that is large enough to encase the target lesion and achieve adequate margin as well as one that is spherical, to minimise non-target parenchyma damage along the axis of the applicator and avoid body-wall burns. This is ideally performed using a single applicator as each additional puncture of the liver capsule increases the risk of bleeding.

While radiofrequency ablation is the most widely used ablative modality, microwave ablation (MWA) can heat through desiccated tissue, achieving larger ablation zones and faster ablation times [4,5]. One criticism of MWA is the ellipsoidal ablation zone produced while RFA with the prongs can produce rounder ablations. One manufacturer (Neuwave, Madison, WI, USA) tackles this by allowing simultaneous use of up to three applicators to achieve a large and round ablation zone. The Emprint system (Medtronic, Boulder, CO, USA) uses Thermosphere^TM^ technology to create a stable field shape around the applicator shaft despite a changing tissue environment to produce large, spherical ablation zones using a single applicator [6].

Each MWA system consists of three components: a generator, cable and antenna(s), each unique with proprietary design. Microwave power is created by the system generator and manufacturers differ in how they specify the generator’s maximum output power. Some manufacturers indicate the power at the front of the generator while others display the estimated power at the applicator tip. Differences in cable design also result in different rates of power loss between the generator and the antenna. Applicators themselves also vary in gauge and are made of various materials. Furthermore, each manufacturer uses their own internally developed ablation performance model to generate the data provided in the ablation-zone dimensional reference charts. For these reasons, a comparison among various manufacturers is challenging due to the lack of standardisation. To date, the current literature comparing ablation geometry between manufacturers in the same setting remains limited. To our knowledge, no study has evaluated the ablation zones of the Solero and Eco MWA systems in an ex vivo porcine liver model. Only one other study has compared the performance of 4 MWA systems in the same setting and that study was published in 2013 on now out-of-date MWA devices [7]. There is a need to study the differences in ablation size and geometry among the state-of-the-art MWA options to guide device selection, especially in a centre where multiple systems are available.

This study compares the ablation zones of four commercially available 2.45 GHz MWA manufacturers (Emprint HP, Eco, Neuwave, Solero) in an ex vivo porcine liver model. These systems were selected as they represent the state-of-the-art MWA technology today.

## 2. Materials and Methods

The first system is the Emprint ablation system consisting of the new Emprint HP ablation generator (CAGENHP) working at 2.45 GHz with a maximum power of 150 W and a reinforced shaft-stiffness perfusion-cooled Emprint Percutaneous Antenna (CA15L2) with Thermosphere Technology (14 G, 15 cm). The second system is the Solero ablation system (AngioDynamics, Latham, NY, USA) consisting of the Solero microwave generator working at 2.45 GHz with a maximum power of 140 W, and a perfusion-cooled antenna (H787700106001US0, 15 G, 14 cm). The third system used is the Eco ablation system (Eco Medical, Nanjing, China) with Eco microwave generator (2.45 GHz) and antenna (14 G, 15 cm). The fourth system used is the Neuwave ablation system (Neuwave, Madison, WI, USA) with Neuwave microwave generator (2.45 GHz) and gas-cooled antennas, PR15 (17 G, 15 cm) and LK15 (17 G, 15 cm).

The primary comparison was among the maximum ablation size that can be created by each of the generator/antenna combinations with a single activation (protocols 1–6). System settings were based on the instructions for use (IFU) provided by each manufacturer, hence comparing the maximum capability of each system with protocols recommended by them. For Eco, a maximum power setting of 120 W was recommended by the manufacturer. The maximum time setting within the IFU was 10 min for all systems with the exception of the Solero system, which has a maximum time setting of 6 min. The secondary comparison was among the ablation size that can be created by each of the generator/antenna combinations at maximum power and a 3 min activation time (protocols 7–12). The goal of the secondary comparison was to evaluate the size and shape of the ablation zones for each generator/antenna combination at a common activation time that may be more applicable for smaller liver lesions.

Ethical approval was not required for this ex vivo animal study. Freshly harvested ex vivo porcine livers (*n* = 85, mean 1.5 kg, range 1.1–2.0 kg) were obtained and sectioned into lobes (left lateral lobe, left medial lobe, right medial lobe and right lateral lobe). Each liver lobe was packaged and sealed in plastic bags, stored in a refrigerator at 6 °C ± 4 °C and used within 24 h. Prior to use, the bagged liver lobes are placed in temperature-controlled water baths set to 22 °C, requiring about 1 h to fully equalize to 22 °C. The intention of this step is to get the tissue to the specified resting ‘room’ temperature, just in time for the ablation sample creation. Before ablation, the liver lobes are removed from the bag and inspected for any damage, discoloration or decay and liver lobes were discarded if such abnormalities were present. Using a calibrated thermometer, the internal resting tissue temperature for each liver lobe is measured and ablation was only performed if the tissue is within 22 °C ± 2 °C.

**Figure 1:**
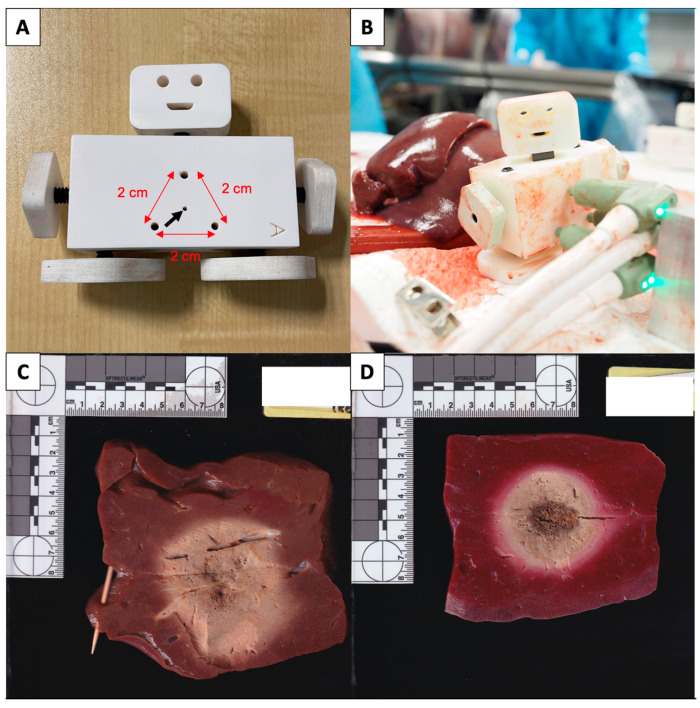
(**A**,**B**) Applicator spacing fixture with 3 holes, 2 cm apart (red arrows), as well as an additional centre hole (black arrow) for the applicator track rod that is used to space the applicators during triple applicator ablations with the Neuwave system. (**C**) Example of a rejected, unstained ablation zone due to a large traversing blood vessel causing distortion of the ablation zone. (**D**) Example of an accepted sectioned and stained ablation zone used for measurement.

For single applicator ablations, the tissue sample is placed on a plastic cutting board, away from any electrically conductive surfaces, which may distort microwave energy. An applicator track tube is placed onto the applicator and the applicator is inserted into the tissue, such that the ablation zone will be optimally centred within the tissue. For triple applicator ablations (protocols no. 6 and 12), an applicator spacing fixture with 3 holes, 2 cm apart, as well as an additional centre hole for the applicator track rod that is used to space the applicators, according to manufacturer IFU (Figure 1A,B). Again, the applicators are placed such that the ablation zone is optimally centred within the tissue.

Once the ablation probes are correctly placed, the ablation protocols are performed. Any interruption of the ablation is recorded. Once the ablation is completed, the applicator track tube is advanced into the ablated tissue for single applicator ablations and the ablation probe is exchanged for a track rod. For triple applicator ablations, the track rod is inserted into the centre hole and the 3 applicators are removed. After the ablated tissue is allowed to rest for 2 min, the ablation is sectioned along the track rod using a sharp blade, revealing 2 halves of the ablation zone. Ablation samples are rejected from the analysis if significantly distorted by crossing vessels or if the dimensions could not be measured due to a lack of unablated tissue around the ablation zone (Figure 1C).

Accepted ablation zones are then stained in 1% triphenyltetrazolium chloride (TTC) solution at 37 °C for 15 min before measurement (Figure 1D). The long-axis diameter (LAD; along the MWA applicator axis) and short-axis diameter (SAD; orthogonal to the MWA applicator axis) measurements of the zones were recorded using a vernier caliper by HGY and MC.

A total of 236 samples were obtained; 32 (13.6%) were rejected for the following reasons: significant distortion of ablation zone by traversing vessel (*n* = 17), insufficient unablated tissue beyond measurement (*n* = 13), and error in ablation system preventing completion of ablation cycle (*n* = 2). A total of 204 samples were included in our analysis. The number of accepted samples for each protocol is illustrated in Table 1.

The geometry of the various protocols was statistically analysed using IBM SPSS Statistics 23. Sphericity index was defined as the ratio between the SAD and LAD. Assuming an ellipsoidal shape, the ablation zone volumes were calculated using the formula V = π × SAD^2^ × LAD/6. Data are described as median and inter-quartile range. Kruskal–Wallis test was used to compare the results. The null hypothesis was rejected when the *p*-value was higher than 0.05.

## 3. Results

### 3.1. Comparison of Maximum System Setting (Protocols 1–6)

**Figure 2:**
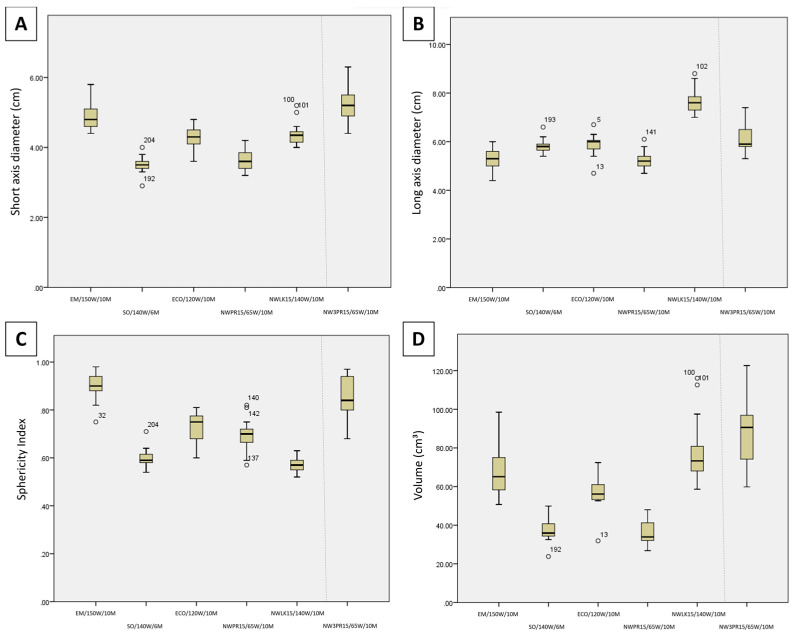
Candlestick chart comparison of the (**A**) short-axis diameter, (**B**) long-axis diameter, (**C**) sphericity index, and (**D**) volume between the maximum system settings of each generator/antenna combination (Protocols 1–6).

Figure 2 illustrates the comparison of SAD, LAD, volume, and SI between the maximum system settings of each generator/antenna combination. Regarding short-axis diameter for maximum system settings (Figure 2A), EM/150 W/10 M had the widest (largest SAD; 4.8 ± 1.4 cm) ablation zones, wider than NWLK15/65 W/10 M (4.4 ± 1.2 cm; *p* = 0.247), ECO/120 W/10 M (4.3 ± 1.2 cm; *p* = 0.194) and significantly wider than NWPR15/65 W/10 M (3.6 ± 1.0 cm; *p <* 0.01), and SO/140 W/6 M (3.5 ± 1.1 cm; *p* < 0.01) among single applicator ablations. NWLK15/65 W/10 M and ECO/120 W/10 M were also significantly wider than NWPR15/65 W/10 M (*p* = 0.017, *p* = 0.029, respectively) and SO/140 W/6 M (*p* = 0.007, *p* = 0.012, respectively). When compared against the three-probe ablation, NW3PR15/65 W/10 M (5.2 ± 1.9 cm) was wider than EM/150 W/10 M (*p* = 0.741), NWLK15/65 W/10 M (*p* = 0.152) and ECO/120 W/10 M (*p* = 0.125) though no significant difference was observed. NW3PR15/65 W/10 M was significantly wider than NWPR15/65 W/10 M (*p* < 0.01) and SO/140 W/6 M (*p* < 0.01).

Regarding long-axis diameter for maximum system settings (Figure 2B), NWLK15/65 W/10 M (7.6 cm) had the longest ablation zones (largest LAD), longer than ECO/120 W/10 M (6.0 cm; *p* = 0.065) and SO/140 W/6 M (5.8 cm; *p* = 0.054), and significantly longer than EM/150 W/10 M (5.3 cm; *p* < 0.001) and NWPR15/65 W/10 M (5.2 cm; *p* < 0.001). NW3PR15/65 W/10 M (5.9 cm; *p* = 0.022) and ECO/120 W/10 M (*p* = 0.044) were also significantly longer than NWPR15/65 W/10 M.

Regarding sphericity index for maximum system settings (Figure 2C), EM/150 W/10 M had the roundest (largest SI, 0.9) ablation zones, significantly rounder than all other single applicator ablation zones, SO/140 W/6 M (0.59; *p* < 0.001), NWLK15/65 W/10 M (0.57; *p* < 0.001), ECO/120 W/10 M (0.75; *p* = 0.01) and NWPR15/65 W/10 M (0.7; *p* < 0.001). NWPR15/65 W/10 M and ECO/120 W/10 M were also rounder than SO/140 W/6 M (*p* = 0.019, *p* = 0.003, respectively) and NWLK15/65 W/10 M (*p* = 0.001, *p* < 0.001, respectively). When compared against the three-probe ablation, EM/150 W/10 M had rounder ablation zones compared with NW3PR15/65 W/10 M (0.84) though no significant difference was observed (*p* = 0.428). NW3PR15/65 W/10 M was significantly rounder than SO/140 W/6 M (*p* < 0.001), NWLK15/65 W/10 M (*p* < 0.001) and NWPR15/65 W/10 M (*p* = 0.014). NW3PR15/65 W/10 M was rounder than ECO/120 W/10 M though no significant difference was found (*p* = 0.102).

Regarding volume for maximum system settings (Figure 2D), EM/150 W/10 M (65.1 cm^3^) and NWLK15/65 W/10 M (73.3 cm^3^) were significantly larger than NWPR15/65 W/10 M (33.9 cm^3^; *p* = 0.010, *p* = 0.002, respectively) and SO/140 W/6 M (median, 35.9 cm^3^; *p* = 0.025, *p* = 0.007, respectively). When compared against the three-probe ablation, NW3PR15/65 W/10 M was larger than EM/150 W/10 M (*p* = 0.402), NWLK15/65 W/10 M (*p* = 0.722) and ECO/120 W/10 M (*p* = 0.135) though no significant difference was observed. NW3PR15/65 W/10 M was significantly larger than NWPR15/65 W/10 M (*p* = 0.001) and SO/140 W/6 M (*p* = 0.004).

### 3.2. Comparison of 3 min Setting (Protocols 7–12)

**Figure 3:**
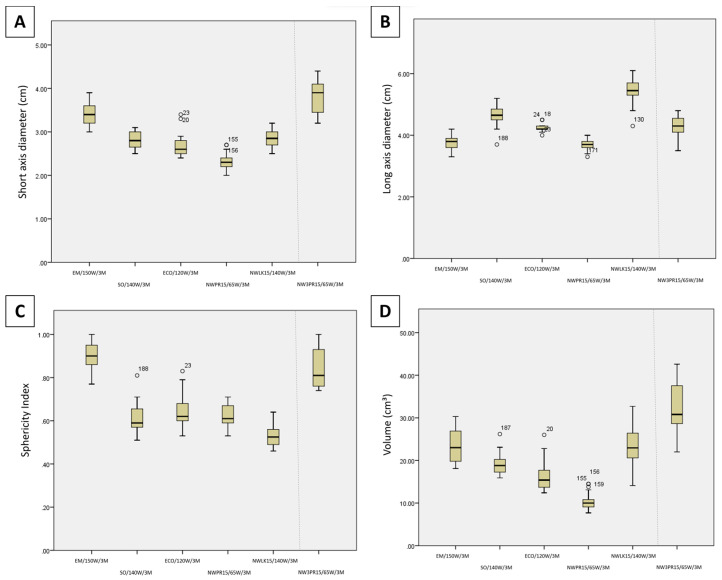
Candlestick chart comparison of the (**A**) short-axis diameter (**B**) long-axis diameter, (**C**) sphericity index, and (**D**) volume between the maximum power 3 min time settings (Protocols 7–12).

Figure 3 illustrates the comparison of SAD, LAD, volume, and SI between each generator/antenna combination at maximum power and 3 min time setting. Regarding short-axis diameter for the maximum power 3 min time settings (Figure 3A), EM/150 W/3 M was significantly wider than NWLK15/65 W/3 M (*p* = 0.010), NWPR15/65 W/3 M (*p* < 0.001), SO/140 W/3 M (*p* = 0.008) and ECO/120 W/3 M (*p* = 0.004). No significant difference was found between NWLK15/65 W/3 M, NWPR15/65 W/3 M, SO/140 W/3 M and ECO/120 W/3 M. When compared against the three-probe ablation, NW3PR15/65 W/3 M was also wider than NWLK15/65 W/3 M (*p* = 0.001), NWPR15/65 W/3 M (*p* < 0.001), SO/140 W/3 M (*p* < 0.001) and ECO/120 W/3 M (*p* < 0.001). No statistical difference was found between SAD of NW3PR15/65 W/3 M and EM/150 W/3 M (*p* = 0.203).

Regarding long-axis diameter for the maximum power 3 min time settings (Figure 3B), NWLK15/65 W/3 M had the longest ablation zones, significantly longer than NWPR15/65 W/3 M (*p* < 0.001), EM/150 W/3 M (*p* < 0.001), SO/140 W/3 M (*p* = 0.018) and ECO/120 W/3 M (*p* = 0.001), as well as NW3PR15/65 W/3 M (*p* = 0.001). SO/140 W/3 M was also found to be significantly longer than NWPR15/65 W/3 M (*p* = 0.003) and EM/150 W/3 M (*p* = 0.004). NW3PR15/65 W/3 M was also significantly longer than NWPR15/65 W/3 M (*p* = 0.047).

Regarding sphericity index for the maximum power 3 min time settings (Figure 3C), EM/150 W/10 M had the roundest (largest SI) ablation zones, significant rounder than all other single applicator ablation zones, SO/140 W/3 M (*p* < 0.001), NWLK15/65 W/3 M (*p* < 0.001), NWPR15/65 W/3 M (*p* < 0.001), ECO/120 W/3 M (*p* < 0.001). SO/140 W/3 M (*p* = 0.048), NWPR15/65 W/3 M (*p* = 0.007) and ECO/120 W/3 M (*p* = 0.006) were each rounder than NWLK15/65 W/3 M. When compared against the three-probe ablation, NW3PR15/65 W/3 M had rounder ablation zones than SO/140 W/3 M (*p* < 0.001), NWLK15/65 W/3 M (*p* < 0.001), NWPR15/65 W/3 M (*p* < 0.001) and ECO/120 W/3 M (*p* < 0.001). EM/150 W/3 M had rounder ablation zones compared with NW3PR15/65 W/3 M, though no significant difference was observed (*p* = 0.387).

Regarding volume for the maximum power 3 min time settings (Figure 3D), EM/150 W/3 M (*p* < 0.001) and NWLK15/65 W/3 M (*p* = 0.002) were each significantly larger than NWPR15/65 W/3 M each, among the single applicator ablations. When compared against the three-probe ablation, NW3PR15/65 W/3 M was significantly larger than SO/140 W/3 M (*p* = 0.006), NWPR15/65 W/3 M (*p* < 0.001) and ECO/120 W/3 M (*p* = 0.001). No significant difference in volume was observed between NW3PR15/65 W/3 M, EM/150 W/3 M and NWLK15/65 W/3 M (Table 2).

All six generator/antenna(s) combinations showed no significant difference between the SI of the maximum time and 3 min time protocols. The SI for the Emprint HP (SI = 0.9) and Solero (SI = 0.59) systems was nearly unchanged as the ablation zone grew in time, in comparison to the Eco and Neuwave systems, which demonstrated lower SI with increased ablation time.

## 4. Discussion

**Figure 4:**
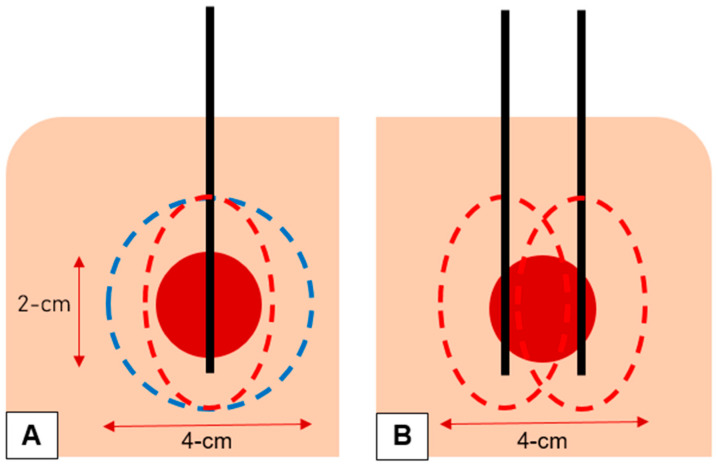
(**A**) A spherical ablation (blue dotted line) is ideal, achieving an even ablative margin around the tumour (red circle) assuming a well-centred antenna (black line) with minimum non-target tissue damage. The inherent ellipsoidal shape of most MWA systems (red dotted line) means it less likely to achieve adequate side-to-side margin while the forward-and-backward margin is reached. (**B**) The ellipsoidal shape of most MWA systems (red dotted line) requires re-positioning of the antenna (black lines) for a 2nd ablation or the use of a 2nd antenna (black lines) to achieve side-to-side margin, resulting in increased time to the procedure or increased risk and cost to the patient, respectively.

To ensure curative ablation and minimize the risk of local tumour progression, a circumferential safety margin of at least 5 mm is paramount to successful ablation [8,9]. In 94 ablated colorectal liver metastases, Wang et al. found that for each 5 mm increase in margin size, a 46% decrease in risk of local tumour progression was achieved [9]. Achieving this minimum 5 mm margin becomes increasingly challenging and increasingly important with increasing tumour size with tumour size being an independent predictor of local tumour progression [9,10,11,12,13,14]. Manufacturers attempt to overcome this by increasing power to increase the volume of the ablation zone; however, the inherent ellipsoidal shape of most MWA systems pose 2 main challenges when ablating a round tumour. Firstly, without adequate width (SAD), obtaining side-to-side ablation margin may not be possible with a single antenna position, requiring overlapping ablations either by re-positioning the antenna or by inserting a 2nd or 3rd antenna into the threatened margin. Secondly, due to the increased length of the ablation zone (LAD), there is an increased risk of non-target tissue damage. A spherical ablation is ideal, achieving an even ablative margin around the tumour assuming a well-centred antenna with minimum non-target tissue damage (Figure 4) [15].

The results of our study show that EM/150 W/10 M produced the most spherical ablation zones in a single applicator at maximum system settings, and is significantly more spherical than SO/140 W/6 M (median, 0.59; *p* < 0.001), NWLK15/65 W/10 M (median, 0.57; *p* < 0.001), ECO/120 W/10 M (median, 0.75; *p* = 0.01) and NWPR15/65 W/10 M (median, 0.7; *p* < 0.001). This finding is commensurate with previous ex vivo as well as clinical studies. Hendriks et al. reported a similar SI of 0.89 when using the Emprint system using various power (60 W and 80 W) and time (3 and 5 min) combinations in an ex vivo porcine liver model [16]. Hendriks et al. also compared the Emprint system with the Amica system and reported significantly more spherical ablations compared with the Amica system (SI = 0.59, *p* < 0.01) [16]. These highly spherical ablations were replicated in a clinical study; Berber reported an SI of 0.9 with the Emprint system, using measurements based on CT performed 2 weeks after MWA in 18 patients who had an average of three tumours with an average tumour size of 1.4 cm [17]. In another clinical study of 53 patients treated with laparoscopic ablation, Zaidi et al. similarly reported SIs of 0.9, 1.0 and 1.1 in three different dimensions using the Emprint system on CT [18]. We also found that EM/150 W/10 M (SI = 0.9) produced more spherical ablation zones compared with NW3PR15/65 W/10 M (SI = 0.84), though no significant difference was found. Using the Neuwave system with three PR15 antennas (2 cm apart), Harari et al. reported a high SI of 0.94 when all three antennas were activated simultaneously at 50 W for 5 min in an ex vivo bovine liver model [19].

At maximum system settings, the single-probe Emprint system produces comparable ablation zones with the simultaneous activation triple-probe Neuwave system. No significant difference in SAD (*p* = 0.741), LAD (*p* = 0.064), SI (*p* = 0.428) or volume (*p* = 0.402) was found between EM/150 W/10 M and NW3PR15/65 W/10 M. Using a single antenna to achieve the desired large spherical ablation zone with the Emprint system would avoid the complexity of placing multiple antennas, reduce the added risk of bleeding and tumour seeding with each antenna insertion and reduce the cost to the patient. That said, one advantage of using multiple probes (2 or 3) would be the ability to have more control over the morphology of the ablation zone, which may be useful when treating tumours with irregular shapes or those close to critical structures.

Our study has several limitations. First, we used an ex vivo non-perfused healthy porcine liver model, hence the ablation zones achieved are likely to be different from ablation zones produced in a clinical setting, e.g., in a patient with liver tumour and background liver cirrhosis. While a live in vivo porcine liver model would be a better surrogate, the high sample size achieved in this study (*n* = 236 before rejection), would not have been feasible using that model. Moreover, estimated ablation zone sizes and geometry using various power and time combinations provided by manufacturers used to guide ablations on the table are mostly based on ex vivo animal models like that utilized in the present study [20]. Second, limited combinations of continuous power and time based upon the IFU provided by the manufacturer were tested, these protocols might not represent the full capabilities of each system. For example, the Neuwave system can deliver short 95W pulses for 1 min in the ‘surgical mode’, which can increase ablation volume and improve sphericity, a protocol not reflected in the IFU [21]. While some have shown larger ablation volumes achieved through the use of power pulsing MWA (high power pulses delivered in intervals), others have shown no meaningful difference in ablation zones achieved using this technique [21,22,23]. Third, this study did not account for the effect of tissue contraction, a known phenomenon in thermal ablation described in pre-clinical studies, though its clinical impact is indeterminate [21,24,25]. Direct measurement of the gross specimen after TTC staining may be underestimating the ‘actual’ ablation zone if there is a significant contraction.

## 5. Conclusions

In conclusion, the new-generation Emprint HP system produced the most spherical ablation zones at maximum system settings and at maximum power for 3 min (SI = 0.9). A spherical ablation zone will increase the chance of achieving an even ablative margin around the tumour while minimizing non-target tissue damage (assuming the target lesion is spherical). At maximum system settings, the Emprint HP system produced relatively large ablation zones, comparable to ablation zones produced by the Neuwave system, which used simultaneous activation of three PR15 antennas, spaced 2 cm apart. Using a single antenna to achieve the desired large spherical ablation zone would avoid the complexity of placing multiple antennas, reduce the risk of bleeding and tumour seeding with each antenna insertion and reduce the cost to the patient.

## Figures and Tables

**Table 1:** Summary of the microwave ablation systems and protocols performed.

No.	Generator	Antenna	Power (W)	Time (min)	Label	*n*
1	Emprint HP CAGENHP	CA15L2, 15 cm	150	10	EM/150 W/10 M	17
2	Solero H78712740000US0	H787700106001US0, 14 cm	140	6	SO/140 W/6 M	15
3	Eco ECO-100E2	ECO-100CL8C, 14 GX15 cm	120	10	ECO/120 W/10 M	15
4	Neuwave NWC1US1N	Neuwave PR15, 15 cm	65	10	NWPR/65 W/1 0M	19
5	Neuwave NWC1US1N	Neuwave LK15, 15 cm	140	10	NWLK/140 W/10 M	16
6	Neuwave NWC1US1N	3× Neuwave PR15, 15 cm	65	10	NW3PR/65 W/10 M	13
7	Emprint HP CAGENHP	CA15L2, 15 cm	150	3	EM/150 W/3 M	25
8	Solero H78712740000US0	H787700106001US0, 14 cm	140	3	SO/140 W/3 M	16
9	Eco ECO-100E2	ECO-100CL8C, 14 G, 15 cm	120	3	ECO/120 W/3 M	14
10	Neuwave NWC1US1N	Neuwave PR15, 15 cm	65	3	NWPR/65 W/3 M	21
11	Neuwave NWC1US1N	Neuwave LK15, 15 cm	140	3	NWLK/140 W/3 M	18
12	Neuwave NWC1US1N	3× Neuwave PR15, 15 cm	65	3	NW3PR/65 W/3 M	15

**Table 2:** Comparison of the ablation sphericity index between the maximum time and 3 min time setting protocols of each system (intra-device).

System	SI of Max	SI of 3 min	*p*-Value
Emprint	0.90	0.90	0.995
Solero	0.59	0.59	0.806
Eco	0.75	0.62	0.066
Neuwave PR15	0.70	0.61	0.090
Neuwave LK15	0.57	0.53	0.363
Neuwave 3× PR15	0.84	0.81	0.985

SI: Sphericity index.

## Data Availability

The data presented in this study are available on request from the corresponding author.
